# Developing and validating a measure of parental knowledge about early math development

**DOI:** 10.3389/fpsyg.2023.1116883

**Published:** 2023-05-18

**Authors:** Ashli-Ann Douglas, Camille Msall, Bethany Rittle-Johnson

**Affiliations:** Department of Psychology and Human Development, Vanderbilt University, Nashville, TN, United States

**Keywords:** home math environment, early math development, patterning knowledge, parent math support, preschool skills, home numeracy development, knowledge of child development

## Abstract

Parents’ knowledge about the math skills that most preschool-aged children can develop might be an important component of the Home Math Environment (HME) as it might shape their math beliefs and efforts to support their preschoolers’ math development. This study aimed to systematically develop measures of parents’ knowledge about two critical early math topics, numeracy, and patterning, across five studies conducted with a total of 616 U.S. parents of 3- to 5-year-olds (66% mothers, 54% sons, 73% White, 60% college-educated). Parents were recruited via CloudResearch or a university database. Study 1 focused on item generation to revise a previous measure to capture a wider set of children’s early math skills and analysis of the psychometric properties of the measure after it was completed by 161 parents via a survey. Study 2 included an analysis of a new sample of parents (*n* = 21) who responded to the measures twice across two weeks to explore test–retest reliability. The measures were iteratively revised, administered to new samples, and analyzed in Studies 3 (*n* = 45), 4 (*n* = 46), and 5 (*n* = 344). The measures demonstrated adequate internal consistency and validity (construct, convergent, and discriminant) in Study 5 such as being positively related to parents’ numeracy and patterning beliefs about their children. Overall, the newly developed measures satisfy standards for the development of an adequate measure and can be used to better understand what parents know about early math development and how this relates to the HME that they facilitate.

## Developing a measure of parental knowledge about early math development

The math support that parents provide their young children at home is predictive of their children’s later math skills (Mutaf-Yıldız et al., 2020). There is wide variability in how often and in what ways parents provide early math support (e.g., Ramani et al., 2015; Susperreguy and Davis-Kean, 2016; Thippana et al., 2020). The expectancy-value theory (EVT) posits that parents’ beliefs affect the academic supports they provide to their children which in turn influence their children’s academic knowledge (Eccles et al., 1983; Jacobs et al., 2004; Eccles and Wigfield, 2020). Additionally, parents’ knowledge about infant development has been theorized to shape their beliefs about and support for their children’s development, including the home literacy environment that they facilitate (Bornstein et al., 2010; Sonnenschein and Sun, 2017). However, while EVT has been extended to include preschool children and the parental math beliefs and support which are relevant for this age group (e.g., Skwarchuk et al., 2014; Douglas et al., 2021), little research on parental math support has focused on parental knowledge about math development. We argue that parents’ knowledge about early math development will help explain variability in their math beliefs and support and be an important addition to theories of parents’ early math support. Indeed, one study has found that among U.S. parents from low- and middle-socioeconomic (SES) backgrounds, parents’ knowledge about early math development was a unique, positive predictor of their children’s math skills (DeFlorio and Beliakoff, 2015).

A few studies have examined the nature of parents’ knowledge about early math development. In one study, most UK mothers of 3- and 4-year-old children misunderstood the nuances of children’s development of an early math skill (Fluck et al., 2005). In particular, most incorrectly anticipated that their child understood aspects of cardinality irrespective of their child’s age and counting abilities. In another study, Canadian parents of 3- to 5-year-olds rated almost all activities on a provided list, including distractor activities like large muscle play, as being “Important” through “Essential” in promoting mathematical development (Skwarchuk, 2009). In the third study, parents from middle SES backgrounds had more accurate knowledge of which math skills were within the developmental range for typical 5-year-olds compared to parents from low SES backgrounds (DeFlorio and Beliakoff, 2015). Notably, parents’ knowledge about early math development was measured in different ways across the three studies and the psychometric properties of the measures were not reported. Further, no study has examined parents’ knowledge about early patterning development, an important component of children’s math development (e.g., Sarama and Clements, 2004; Fyfe et al., 2019). Given the potential role of parents’ knowledge about early numeracy and patterning development for the home math support that they provide, reliable and valid measures of their knowledge are needed. The current study aimed to validate a measure of parents’ knowledge about early math development.

## General method

A closed-ended, self-report measure of parents’ knowledge about early math development was iteratively revised and administered electronically to 616 U.S. parents of 3- to 5-year-olds across five studies in 2021 and 2022. Parents were recruited via CloudResearch for each study except for study 2 in which parents were recruited via a university department database. For each study, parents were paid $1.80 to $10 for participating. Participant demographics and descriptive statistics for each study are reported in Tables 1, 2 respectively.

**Table 1:** Demographic statistics of participants by study.

Variable	Frequencies (%)	Study 1(*N* = 161)	Study 2(*N* = 21)	Study 3(*N* = 45)	Study 4(*N* = 45)	Study 5(*N* = 344)
**Mothers**	52	86	67	71	56
**Primary Caregiver**	–	–	87	89	94
Child Age					
3-year-olds	30	–	35	31	52
4-year-olds	34	100	63	69	48
5-year-olds	36	–	–	–	–
**Sons**	55	57	49	47	61
**Income**					
Less than $45,000	–	5	29	31	28
$45,000 - $89,999	–	19	42	42	41
More than $90,000	–	76	29	27	32
**Education**					
Less than a Bachelor’s	34	–	44	56	21
Bachelor’s Degree	45	43	31	31	55
More than a Bachelor’s	21	57	24	13	24
**Race/Ethnicity**					
White	75	76	67	69	77
Black	12	11	11	11	8
Asian or Pacific Islander	8	5	4	7	5
Biracial or Mixed Race	4	8	16	7	4
American Indian or Native or Other Race	1	–	–	6	6

**Table 2:** Descriptive statistics of knowledge measure subscales across studies (average accuracy and standard deviations).

Measure	Study 1	Study 2		Study 3	Study 4	Study 5
		Time 1	Time 2			
Across all items	0.75 (0.12)	0.77 (0.10)	0.80 (0.10)	0.78 (0.07)	0.79 (0.08)	0.68 (0.12)
Numeracy	0.74 (0.17)	0.78 (0.13)	0.81 (0.15)	0.76 (0.14)	0.78 (0.13)	0.65 (0.17)
Within	0.82 (0.22)	0.88 (0.17)	0.85 (0.20)	0.85 (0.16)	0.80 (0.19)	0.79 (0.23)
Beyond	0.55 (0.39)	0.54 (0.40)	0.71 (0.34)	0.56 (0.38)	0.72 (0.35)	0.38 (0.37)
Pattern	0.76 (0.16)	0.79 (0.14)	0.82 (0.18)	0.80 (0.12)	0.81 (0.13)	0.68 (0.17)
Within	0.78 (0.20)	0.81 (0.20)	0.84 (0.27)	0.80 (0.18)	0.80 (0.18)	0.74 (0.24)
Beyond	0.70 (0.35)	0.78 (0.29)	0.78 (0.30)	0.81 (0.29)	0.84 (0.29)	0.54 (0.41)

^a^Study 4 and 5 included two additional patterning within items. ^b^Study 5’s mean excludes two items: numeracy within item 5 and pattern within item 10.

## Study 1

Study 1 aimed to (1) develop a more comprehensive measure of parents’ knowledge about early math development through item generation and (2) examine the refined measure’s internal consistency and content and construct validity. The measure was administered to 161 parents of 3- to 5-year-old children via a survey after being revised. Parents also completed a survey about their math and literacy beliefs.

### Beliefs about child math and literacy abilities

The parental beliefs survey was composed of items adapted from a previous instrument (Zippert and Rittle-Johnson, 2020). Specifically, they were asked, “How good is your child currently in each area listed below?.” They reported about two numeracy items (i.e., “Counting and naming numbers” and “Comparing the magnitudes (size) of numbers”), two patterning items (i.e., “Noticing and making patterns” and “Figuring out what should come next in patterns”), and one literacy item “Learning to read and write.” Their ratings of the two numeracy and two patterning items were averaged as measures of their perception of their child’s numeracy and patterning abilities, respectively.

### Knowledge about early math development survey

The measure was adapted from a previously used measure (DeFlorio and Beliakoff, 2015) whose instruction was “These following questions concern children’s mathematical development during the preschool years. Which of the following abilities or skills do you believe typical children have developed before their 5th birthday?.” The previously used measure had a dichotomous scale and used 23 items. It included 13 items on numeracy skills, with six items on skills that are within the typical developmental range for most five-year-olds and seven that are beyond their typical developmental range. The previous measure also included two items on patterning skills (both within the developmental range) and eight items on spatial skills (four within and four beyond).

#### Item generation and content validity

We discuss how we generated or revised items for the measure and how the items relate to the literature on early math development as evidence of the measure’s content validity (Joint Committee on the Standards for Educational and Psychological Testing, 2014). Our goal was to have reliable subscales of early math domains, given previous research indicating that preschoolers can and should be learning about early math skills including patterning and spatial skills (Verdine et al., 2014; Fyfe et al., 2019). Thus, we created items to measure a wider variety of early math skills given that more than half of the items in the original measure focused on numeracy. For example, we created a new item, “Fill in the missing part of a pattern made of repeating objects (for example, circle, square, square, circle, square, _____, circle, square, square)” based on Rittle-Johnson et al. (2020) in order to include more items on patterning skills. In general, we referenced prior research on early math development to identify math tasks most children in the U.S. are able to accomplish independently before kindergarten or their fifth birthday (e.g., National Research Council, 2009; Claessens and Engel, 2013; Clements and Sarama, 2014; Rittle-Johnson et al., 2017, 2019; Litkowski et al., 2020a; Kaufman et al., 2021). See Supplementary Table S1 for details about the final measure’s items including the origin of each item.

Overall, sixteen items (8 patterning, 5 numeracy, and 3 spatial) were inductively added to measure a wider variety of math skills and to make the measure similar across subscales. Nine items were dropped primarily because they were ambiguous or were very similar to other items (e.g., “Use a computer with age-appropriate software to learn math concepts” did not focus on a specific numeracy, patterning, or spatial skill). Ten items from the original measure were also revised for clarity. The instruction was also expanded with the addition of a sentence that reads “Please select ‘yes’ for each skill that you think most children in the United States correctly master by age five. Please select ‘no’ for each skill that you do not think most children in the United States correctly master by age five.”

After the first round of edits, the measure included 30 items (10 numeracy, 10 patterning, and 10 spatial). Within each subscale, there were seven items on skills children typically develop by age five (within the developmental range) and three on skills children typically do not develop by age five (beyond the developmental range). Parents’ correct responses to each item were scored as a 1 and incorrect answers were scored as a 0. Parents’ scores were averaged to create a measure of their knowledge across numeracy, patterning, and spatial. Parents’ scores were also averaged to create separate composite measures for numeracy, patterning, and spatial for their knowledge (1) across all items, (2) about skills within the typical developmental range for most preschoolers, and (3) about skills beyond the typical developmental range for most preschoolers.

### Results

#### Reliability

We used Cronbach’s alpha as an indicator of internal consistency and interpreted alpha levels based on previous research (Cooper and Schindler, 2003; Cohen et al., 2007). The measure had moderate/acceptable internal consistency across all items (*α* = 0.60) but had unacceptable reliability when considering subscales composed of all numeracy (*α* = 0.36), patterning (*α* = 0.32), and spatial (*α* = −0.02) items. When considering only the items that are within the typical development range for preschoolers, the numeracy subscale had moderate/acceptable reliability (*α* = 0.69), the patterning subscale had low reliability (*α* = 0.53) and the spatial subscale had unacceptable (but much better) reliability (*α* = 0.45). When considering the items that are beyond the typical development range for preschoolers, both the numeracy subscale (*α* = 0.69) and patterning subscales (*α* = 0.67) had moderate/acceptable reliability while the spatial subscale had unacceptable reliability (*α* = 0.46). Given the poor reliability of the spatial subscales, we focused further analyses and revisions on the numeracy and patterning subscales.

#### Construct validity

We used Confirmatory Factor Analyses (CFA) to examine whether each subscale’s items measured a shared construct as indicated by each item having a significant factor loading. All numeracy items loaded significantly onto a model with two correlated factors, with one factor including items that measure skills that are beyond the typical developmental range for preschoolers and the other factor including items that measure skills that are within the developmental range (see standardized factor loadings in Table 3). All patterning items except for item 5 (“Sort a set of objects into 3 groups based on color such as red, blue, and green”) loaded significantly onto a similar 2-factor model (see standardized factor loadings in Table 4). Thus, we concluded that we have evidence of construct validity: all numeracy items do measure the same construct and most patterning items do measure the same construct.

**Table 3:** Descriptive statistics for measure of parents’ knowledge about early numeracy development within the developmental range.

Item	Study 1 (*N* = 161)		Study 2 (*N* = 21)	Study 3 (*N* = 45)	Study 4 (*N* = 45)	Study 5 (*N* = 344)	
	*M* (SD)	Factor Loading^a^	*M* (SD)	*M* (SD)	*M* (SD)	*M* (SD)	Factor Loading^a^
Count a row of 15 objects (for example, count 15 plastic worms) ^a^	0.96 (0.19)	0.29	0.89 (0.31)	1.00 (0)	0.93 (0.25)	0.94 (0.23)	–
Counts out the correct number of things when asked for a specific number of things up to 10 (for example gives 6 cookies when asked for 6 cookies)	0.81 (0.39)	0.68	92 (0.28)	0.93 (0.25)	0.91 (0.28)	0.89 (0.32)	0.39
Name the written numbers from 1 to 10 (for example, points to the 9 when asked “where is the number nine?”)	0.87 (0.34)	0.48	97 (0.16)	0.89 (0.32)	0.87 (0.34)	0.86 (0.35)	0.50
Solve small addition or subtraction problems presented with objects (for example, 3 blocks and 2 blocks is ___ blocks)	0.73 (0.45)	0.51	84 (0.37)	0.73 (0.45)	0.76 (0.43)	0.82 (0.39)	0.30
Tell which of two spoken numbers between one and ten is bigger (for example, says “five” in response to “Which is bigger, five or two?”)	0.86 (0.34)	0.51	92 (0.28)	0.93 (0.25)	0.78 (0.42)	0.76 (0.43)	0.58
Tell which of two written numbers between one and ten is bigger (for example, points to the written number 9 when shown the written numbers 2 and 9 and asked “Which is bigger”)	0.86 (0.34)	0.52	89 (0.31)	0.84 (0.37)	0.72 (0.46)	0.76 (0.43)	0.54
Answer questions by adding or subtracting small numbers (for example, says “three” in response to “If you have four stickers and then you give me one of your stickers, how many stickers would you have left?”)	0.65 (0.48)	0.44	70 (0.46)	0.60 (0.50)	0.63 (0.49)	0.68 (0.47)	0.35

a Factor loadings are standardized.

**Table 4:** Descriptive statistics for measure of parents’ knowledge about early numeracy development within the developmental range.

Item	Study 1 (*N* = 161)		Study 2 (*N* = 21)	Study 3 (*N* = 45)	Study 4 (*N* = 45)	Study 5 (*N* = 344)	
	*M* (SD)	Factor Loading	*M* (SD)	*M* (SD)	*M* (SD)	*M* (SD)	Factor Loading^a^
Continue a pattern of cubes (for example, blue, blue, red, red, blue, blue, red, red, __, __, __, _)	0.83 (0.31)	0.56	0.81 (0.40)	0.76 (0.43)	0.80 (0.40)	0.76 (0.43)	0.42
Use colored beads to make a simple pattern, such as a “blue-purple” pattern	0.88 (0.32)	0.37	0.92 (0.28)	0.91 (0.29)	0.85 (0.36)	0.78 (0.42)	0.34
Figure out what should come next in a simple pattern (for example: clap, stomp, clap, stomp, _, _)	0.85 (0.36)	0.34	0.84 (0.37)	0.98 (0.15)	0.87 (0.34)	0.72 (0.45)	0.45
Sort a set of objects into 2 groups based on color such as red and blue	0.91 (0.29)	0.19	1.00 (0)	1.00 (0)	93 (0.25)	91 (0.29)	–
Identify two patterns that follow the same rule made with different materials (for example, a block-block-ball pattern and a sun-sun-moon pattern are similar)	0.65 (0.48)	0.29	0.58 (0.50)	0.56 (0.50)	0.54 (0.50)	0.60 (0.49)	0.45
Fill in the missing part of a pattern made of repeating objects (for example: circle, square, square, circle, square, __, circle, square, square)	0.76 (0.43)	0.66	86 (0.35)	0.71 (0.46)	0.67 (0.47)	0.63 (0.48)	0.61
Make the same kind of simple pattern in their bracelet as their friends’ bracelet, but using different colors (for example, your child makes a yellow-green pattern to match a friend’s red-blue pattern)	0.60 (0.49)	0.24	0.69 (0.47)	0.67 (0.48)	0.78 (0.42)	0.65 (0.48)	0.38
Makes a repeating pattern (for example, makes a clap, spin, snap, clap, spin, snap pattern)	NA	NA	NA	NA	0.85 (0.36)	0.74 (0.44)	0.50
Copy a pattern someone else makes in the same way (for example, your child beats a drum in a loud-soft pattern just like do)	NA	NA	NA	NA	0.89 (0.31)	0.80 (0.40)	0.33

a Factor loadings are standardized.

We also compared the 2-factor models to 1-factor models which did not consider items measuring skills that are within the typical developmental range separately from items that measure skills that are beyond the typical developmental range. We found that the 2-factor model fit the data significantly better than a 1-factor model for both numeracy, χ^2^(3) = 98.28, *p* < 0.001, and patterning, χ^2^(3) = 31.7, *p* < 0.001. This finding suggests that the subscales (within the typical developmental range for preschoolers and beyond the typical developmental range for preschoolers) were unique. The subscales (within the typical developmental range for preschoolers and beyond the typical developmental range for preschoolers) were negatively correlated providing additional evidence that they were unique and needed to be treated as separate measures, *r*_numeracy_(159) = −0.25, *p* < 0.001 and *r*_pattern_(159) = −0.33, *p* < 0.001. Further studies focused on revising and validating the subscales consisting of items that are within the developmental range for most typically developing preschool-aged children given that the intended audience of the measure was parents of preschoolers. Additionally, the subsequent studies focused on validating the subscales among parents of 3-year-olds and/or 4-year-olds since the measures ask about skills that are relevant to preschool-aged children who are younger than 5 years old.

## Study 2

Study 2 aimed to examine the internal consistency as well as test–retest reliability of the measures of parents’ knowledge about early numeracy and patterning development with a new sample. The items were identical to Study 1, however, the question stem was revised to ask about “most children” instead of “typical children.” The measure was administered twice across two weeks to 21 parents of 4-year-old children. Parents were randomly assigned to a condition and received information about early numeracy or patterning skills between the sessions; however, analyzing the effect of this information is beyond the scope of this report. Additionally, to examine the clarity of the measure, a subset of parents (*n* = 7) completed a video-recorded think-aloud (van Someren et al., 1994) as they completed the measures. Specifically, they were told, “We want to understand how parents approach answering our questions. Please read the following questions aloud and think aloud while you decide which answer you will choose. Keep talking as you answer all the questions and try to say everything that goes through your mind.”

### Results

#### Reliability

In contrast to study 1, and perhaps due to the much smaller sample size, the measure’s internal consistency was low for the items measuring numeracy skills within the typical developmental range for preschoolers (α = 0.53) and moderate for the items measuring patterning skills within the typical developmental range (α = 0.64) at Time 1. Notably, both subscales had high reliability at Time 2 after parents received some information designed to change their knowledge about early numeracy or patterning development (α_numeracy_ = 0.72 and α_patterning_ = 0.87). Additionally, these subscales had high test–retest reliability, with strong correlations between the numeracy scores, *r*(19) = 0.65, *p* = 0.001, and patterning scores, *r*(19) = 0.80, *p* < 0.001, across two weeks.

#### Think aloud

Two authors made notes about parents’ comments and questions during the think-aloud sessions and identified themes that emerged among parents after discussing their notes. First, several parents posed clarifying questions to the experimenter as they participated in the think-aloud. Overall, parents asked nine questions as they read and responded to the knowledge measure. This included questions about the difference between “numerals” and “numbers” which suggested that some items’ phrasing needed to be clarified as well as uncertainty about what was meant by “most children” in the knowledge measure question. We revised the measure based on this feedback. Second, many parents expressed being unsure about their answers to knowledge measure items. Parents frequently said “I do not know” (25 times) or “probably” (10 times) when thinking through their answers to individual items. This finding suggests that parents have little confidence in their knowledge about early math skills.

## Studies 3 and 4

Studies 3 and 4 aimed to revise the measure based on the previous two rounds of data collection and examine the internal consistency of the revised measure. As such, the revised measure was administered to two new samples each including 45 parents.

### Knowledge measure revisions

We examined item-total correlations, standardized factor loadings, and means and standard deviations of each item in Studies 1 and 2 to identify items that needed to be revised. Specifically, we flagged and revised items with item-total correlations less than 0.2 and non-significant factor loadings and items that over 95% of parents responded to correctly or incorrectly. One numeracy item was revised to increase its difficulty (from counting 10 objects to counting 15 objects) given that it was previously flagged twice for evidence of a ceiling effect (M = 0.96, SD = 0.19 in Study 1 and M = 0.89, SD = 0.31 in Study 2). Two patterning items about skills within the developmental range were added in case any patterning items needed to be dropped in further analyses given that five patterning items were flagged at least once for item-total correlations less than 0.2, nonsignificant factor loadings, and ceiling effect. The new version of the measure included 32 items.

Based on parents’ questions during Study 2’s think-aloud, we made changes to the phrasing of 13 items. For example, we changed “numerals” to “written numerals,” added “spoken” at the beginning of phrases about “numbers” referencing numbers heard aloud, and removed “alternating” from the phrase “simple alternating pattern.” We also modified the instruction to “Please select “Yes” for each skill that you think over 50% of children in the United States can correctly do by their 5th birthday. Otherwise, select “No.”” This decision to specify “over 50%” was made based on some parents’ comments that they were unsure what “most children” meant in study 2. Fifty percent has been used as a cutoff in previous literature to indicate proficiency levels (Claessens and Engel, 2013; Litkowski et al., 2020b).

Following Study 3, five numeracy and four patterning items were revised. The revisions included changes to wording to increase the clarity of seven items as well as to increase the difficulty of 2 items (e.g., “name the numerals from 1 to 5” was changed to “name the written numbers from 1 to 10”).

### Results

When considering the items that are within the typical development range for preschoolers, both the numeracy subscale (α = 0.45) and patterning subscale (α = 0.49) had unacceptable reliability in Study 3. The numeracy subscale continued to have unacceptable reliability in Study 4 (α = 0.44), but the patterning subscale’s scale reliability improved slightly (α = 0.52).

## Study 5

Study 5 aimed to analyze the knowledge measure with a larger sample using a pre-registered analytic plan.^1^ Notably, the larger sample allowed for analyses like Confirmatory Factor Analyses. A secondary aim of study 5 was to understand how parents approached the measure and potential sources of their knowledge about early math development. As such immediately after the knowledge measure items, parents were asked to type a response to the question “Overall, how did you decide which answers to choose when deciding which academic skills most children in the United States develop by age five?”

### Results

#### Reliability

Items focused on numeracy skills that are within the developmental range had low (but not unacceptable) reliability (α = 0.59). The Kuder–Richardson Formula 20 (KR20), often considered a better measure of reliability for tests with dichotomous variables, yielded a similar estimate (0.60). One item (“Count a row of 15 objects”) had a low item-total correlation. As such, it was excluded from further analyses. The patterning items that are within the developmental range had moderate/acceptable reliability (α = 0.65 and KR20 = 0.66). As with patterning, one item (“Sort a set of objects into 3 groups based on color such as red, blue, and green”) had a low item-total correlation and was excluded from further analyses.

#### Construct validity

We used CFA to examine whether each subscale’s items measured a shared construct as indicated by each item having a significant factor loading (see standardized factor loadings in Tables 3, 4). All numeracy items measuring skills within the typical developmental range for most preschoolers loaded significantly onto a factor. Similar to study 2, all patterning items measuring skills within the typical developmental range except for item 5 loaded significantly onto a factor. Importantly, the models fit the data well according to several indices such as a nonsignificant chi-square, Comparative Fit Index >0.9, Root Mean Square Error of Approximation <0.08, and Adjusted Goodness of Fit >0.9 (see Supplementary Table S2). Thus, we concluded that after dropping item 5, we have evidence of construct validity: numeracy items do measure the same construct and patterning items do measure the same construct.

#### Convergent validity

Next, we measured the convergent validity of the numeracy and patterning subscales. The Joint Committee on the Standards for Educational and Psychological Testing (2014, p. 16–17) reports that “relationships between test scores and other [external] measures intended to assess the same or similar constructs provide convergent evidence.” We view parents’ knowledge and beliefs as similar constructs- two types of parental cognitions that potentially influence their support. We examined whether parents’ knowledge about numeracy skills that most children develop by age 5 was related to their beliefs about their child’s numeracy ability and did the same for patterning. We found evidence of convergent validity for both. Specifically, parents’ knowledge about early patterning development was significantly correlated with their perception of their child’s patterning abilities, *r*(342) = 0.24, *p* < 0.001. Parents’ knowledge about early numeracy development was also significantly correlated with their perception of their child’s numeracy abilities, *r*(342) = 0.11, *p* = 0.036.

#### Discriminant validity

In line with research indicating that the home numeracy environment and the home literacy environment are separate constructs (even though they have some shared variance; Napoli and Purpura, 2018), we view parental *belief about literacy* as a distinct construct from their knowledge about math. As such, we examined the relationship between these two constructs as an indicator of discriminant validity. We found evidence of discriminant validity for both types of parental knowledge. Specifically, parents’ knowledge about early numeracy development was not correlated with their belief about their child’s literacy abilities, *r*(343) = 0.05, *p* = 0.342. Additionally, parents’ knowledge about early patterning development was not correlated with their perception of their child’s literacy abilities, *r*(343) = 0.09, *p* = 0.112.

#### Parent-reported sources of their knowledge about early math development

To explore potential sources of parents’ knowledge about early math development, we examined their reports of how they decided on their answers (see Supplementary Table S2 for additional details). Forty-three percent of parents’ responses did not fit the coding scheme (e.g., some parents responded to the question with a single word). Of the 196 parents whose responses were coded, most mentioned that they thought about their experience with other children (54%) and/or their perception of their participating child’s current abilities and their expectations for their participating child’s math development (45%). Very few parents mentioned their knowledge of benchmarks or other information about children’s early math development (16%).

## Discussion and implications

The current study contributes to the development of a measure of early math development for preschool-aged children. As far as the authors know, this is the first paper to iteratively revise and develop a measure of parents’ knowledge of early math development. Prior research shows there is variability in the frequency and complexity of math activities that parents engage in with their preschool-age children (e.g., Zippert and Rittle-Johnson, 2020). One potential source of this variability is parents’ knowledge of early math development (Douglas, 2022). The current paper provides evidence for the internal consistency, test–retest reliability, and validity of the measure of parents’ knowledge of early numeracy and patterning development among mostly college-educated, White US parents of 3- to 5-year-olds. Specifically, we had evidence of construct validity across two studies: numeracy items measured the same construct, and patterning items measured the same construct. Additionally, the final version of the knowledge measure demonstrates strong evidence for construct, convergent, and discriminant validity and some evidence of reliability for the numeracy and patterning subscales.

### Limitations

Despite five rounds of measurement development and pilot testing, a limitation of the current study was that the measures of parents’ knowledge about early numeracy and patterning development were only somewhat reliable, suggesting that additional measure development research could be beneficial. Additionally, we focused on validating separate subscales rather than a “math” measure. Notably, in Study 2, we found that the measures were substantially more reliable after parents received information about early math development. The inclusion of a related intervention between the time periods is uncommon for examining test–retest reliability; however, the evidence of high test–retest reliability suggests that the internal consistency of the measures was stable across the time points *despite* the information that parents received and that the measures can be used as predictors. Another limitation could be the dichotomous nature of the answer choices. Parents were given the option to choose “yes” or “no” to each item, but parents could have been unsure or wanted to choose “maybe.” This decision may bias the results of our measure. Relatedly, while we noted how often parents who did the Think Aloud expressed uncertainty while completing the measure, we did not systematically measure parents’ confidence or include a measure of parents’ confidence in scoring their knowledge. Parents’ low confidence in their knowledge about some skills could have reduced measure reliability. Finally, while we had some successes with recruiting a diverse sample (e.g., some studies included almost 50% of fathers and parents from almost all US states), most parents were White, had at least a Bachelor’s degree, and had a household income of at least $45,000. This limits the generalizability of our findings.

## Conclusion

Overall, the current study furthers the development and validation of a new measure relevant to the Home Math Environment (HME) which includes the math-related interaction, attitudes, and beliefs facilitated by parents of preschoolers. We provide details on a reliable and valid measure of parents’ early numeracy knowledge that satisfies standards for the development of an adequate measure (Cooper and Schindler, 2003; Cohen et al., 2007; Joint Committee on the Standards for Educational and Psychological Testing, 2014). This allows for further research on the role parents’ knowledge about early math development may play in their efforts to support their children’s math development and sources of parents’ knowledge about early math development. Such research could potentially inform interventions to support family members in providing meaningful and appropriate math learning experiences at home to support their children’s math development and readiness.

## Data availability statement

The raw data supporting the conclusions of this article will be made available by the authors, without undue reservation.

## Ethics statement

The studies involving human participants were reviewed and approved by Vanderbilt University Institutional Board. The patients/participants provided their written informed consent to participate in this study.

## Author contributions

A-AD: project conceptualization, design, admin, data collection, data analysis, writing, reviewing, and revision. CM: project design–specifically measure revision, data collection, data analysis, writing, reviewing, and revision. BR-J: project conceptualization, design, admin, supervision, writing, and reviewing. All authors contributed to the article and approved the submitted version.

## Funding

This study was supported by Heising Simons Foundation 2021-2773.

## Conflict of interest

The authors declare that the research was conducted in the absence of any commercial or financial relationships that could be construed as a potential conflict of interest.

## Publisher’s note

All claims expressed in this article are solely those of the authors and do not necessarily represent those of their affiliated organizations, or those of the publisher, the editors and the reviewers. Any product that may be evaluated in this article, or claim that may be made by its manufacturer, is not guaranteed or endorsed by the publisher.

## Footnotes

^1^ https://aspredicted.org/2YG_39S
