# Supplementary material for: Developing and validating a measure of parental knowledge about early math development
Source: Front Psychol. 2023 May 18;14:1116883. doi: 10.3389/fpsyg.2023.1116883 (PMC10232842; doi:10.3389/fpsyg.2023.1116883)
Supplement: Supplementary file 1 [file Data_Sheet_1.PDF]

Table S1

Details About the Measure of Parents' Knowledge about Early Math Development used in Study 5

Order	Item	Specific Skill	Learning Trajectory Level	Question Origin	Previous Version
**Numeracy**					
**Within**					
1	Count a row of 15 objects (for example, count 15 plastic worms)	Object Counting/ One to One Correspondence	Counter (10)	Deflorio & Beliakoff (2015)<br>Modified based on previous research (Clements et al., 2014; Litkowski et al., 2020	Count a row of 10 objects (for example, count ten plastic worms)
13	Counts out the correct number of things when asked for a specific number of things up to 10 (for example, gives 6 cookies when asked for 6 cookies)	Cardinality	Counter And Producer (10+)	Created based on previous research (e.g., Clements et al., 2014; Litkowski et al., 2020)	Give the correct number of things when asked for a specific number of things up to 10 (for example, gives 6 cookies when asked for 6 cookies)
14	Name the written numbers from 1 to 10 (for example, points to the 9 when asked "where is the number nine?")	Numeral ID	Functional Numeral User	Deflorio & Beliakoff (2015)	Name the numerals from 1 to 5 (for example, points to the 1 when asked "where is the numeral one?")
16	Solve small addition or subtraction problems presented with objects (for example, 3 blocks and 2 blocks is ___ blocks)	Simple Arithmetic	Find Result +-	Deflorio & Beliakoff (2015)	Solve small addition or subtraction problems presented with objects, such as 3 blocks and 2 blocks = __ blocks
23	Tell which of two spoken numbers between one and ten is bigger (for example, says "five" in response to "Which is bigger, five or two?")	Symbolic Magnitude Comparison	Counting Comparer (10)	Created based on previous research (e.g., Clements et al., 2014)	Tell which of two numbers between one and ten is bigger (for example, says "five" in response to "Which is bigger, five or two?")

25	Tell which of two written numbers between one and ten is bigger (for example, points to the written number 9 when shown the written numbers 2 and 9 and asked "Which is bigger")	Symbolic Magnitude Comparison	Counting Comparer (10)	Created based on previous research (e.g., Clements et al., 2014)	Tell which of two numerals between one and ten is bigger (for example, points to the numeral 9 when shown the numerals 2 and 9 and asked “Which is bigger")
29	Answer questions by adding or subtracting small numbers (for example, says "three" in response to "If you have four stickers and then you give me one of your stickers, how many stickers would you have left?")	Simple Arithmetic	Find Result +-	Created based on previous research (e.g., Clements et al., 2014; Litkowski et al., 2020)	Answer questions by adding or subtracting small numbers (for example, says "three" in response to "If you have four stickers. How many stickers would you have if you give me one sticker)
**Beyond**					
8	Solve single-digit addition or subtraction problems presented on flashcards (for example, 5 + 3 = ___)	Advanced Arithmetic	Numbers In Numbers +/-	Deflorio & Beliakoff (2015)	Solve single-digit addition or subtraction problems presented on flashcards, such as 5 + 3 = __
11	Recite number words from 1 to 100	Rote Counting - 100	Counter To 100	Deflorio & Beliakoff (2015)<br>Modified based on previous research (Litkowski et al., 2020)	Recite numbers from 1 to 10
27	Name the written numbers from 1 to 100 (for example, says the word "ninety-three" when shown the written number 93)	Numeral ID - 100	Decade Number Identifier	Created based on previous research (e.g., Clements et al., 2014; Litkowski et al., 2020)	Name the numerals from 1 to 100 (for example, says the word "ninety-three" when shown the written number 93)
			**Pattern**		
**Within**					
2	Continue a pattern of cubes (for example, blue, blue, red, red, blue, blue, red, red, _______, _______, _______, _______)	Extend	Pattern Extender	Created based on previous research (e.g., Clements et al., 2014; Kaufman et al., 2021;	NA

				Rittle-Johnson et al., 2020)	
3	Use colored beads to make a simple pattern, such as a "blue-purple" pattern	Create	Patterner	Deflorio & Beliakoff (2015)	Use colored beads to make a simple pattern, such as "blue-purple"
4	Figure out what should come next in a simple pattern (for example: clap, stomp, clap, stomp, _______, _______)	Extend	Pattern Extender AB	Created based on previous research (e.g., Clements et al., 2014; Kaufman et al., 2021; Rittle-Johnson et al., 2020)	Figure out what should come next in a simple alternating pattern (for example: clap, stomp, clap, stomp, _______, _______)
5	Sort a set of objects into 3 groups based on color such as red, blue, and green	Sort/Similarities Differences	Pre-Explicit Patterner 2	Created based on previous research (e.g., Clements et al., 2014; Kaufman et al., 2021; Rittle-Johnson et al., 2020)	Sort a set of objects into 2 groups based on color such as red and blue
10	Identify two patterns that follow the same rule made with different materials (for example, a block-block-ball pattern and a sun-sun-moon pattern are similar)	ID Rule	Pattern Unit Recognizer	Created based on previous research (e.g., Clements et al., 2014; Kaufman et al., 2021; Rittle-Johnson et al., 2020)	NA
18	Fill in the missing part of a pattern made of repeating objects (for example: circle, square, square, circle, square, _____, circle, square, square)	Missing	Pattern Fixer	Created based on previous research (e.g., Clements et al., 2014; Kaufman et al., 2021; Rittle-Johnson et al., 2020)	NA
22	Make the same kind of simple pattern in their bracelet as their friend's bracelet, but using different colors (for example, your child	Abstract	Pattern Unit Recognizer	Created based on previous research (e.g., Clements et al., 2014; Kaufman et al., 2021;	Make the same kind of simple alternating pattern in their bracelet as their friend's bracelet, but using different colors (for example, your child makes a yellow-green

	makes a yellow-green pattern to match a friend's red-blue pattern)			Rittle-Johnson et al., 2020)	pattern to match a friend's red-blue pattern)
31	Makes a repeating pattern (for example, makes a clap, spin, snap, clap, spin, snap pattern)	Create	Patterner	Created based on previous research (e.g., Clements et al., 2014; Kaufman et al., 2021; Rittle-Johnson et al., 2020)	NA
32	Copy a pattern someone else makes in the same way (for example, your child beats a drum in a loud-soft pattern just like you do)	Copy	Pattern Duplicator	Created based on previous research (e.g., Clements et al., 2014; Kaufman et al., 2021; Rittle-Johnson et al., 2020)	NA
**Beyond**					
12	Use number patterns to solve problems (for example, fills in the blanks when presented with 26, 22, 18, __, 10, __)	Growing ID Pattern Rule/Unit	Beginning Arithmetic Patterner	Created based on previous research (e.g., Clements et al., 2014)	NA
20	Figure out what comes next in a growing pattern that subtracts two cubes for each stair in a staircase (for example, 9 cubes, 7 cubes, 5 cubes, 3 cubes, ____)	Growing Extend	Pattern Unit Recognizer	Created based on previous research (e.g., Clements et al., 2014; Kaufman et al., 2021; Rittle-Johnson et al., 2020)	NA
30	Describe even numbers (for example, 2, 4, 6, 8, 10) as “skipping” every other number on a 100s chart	Growing ID Pattern Rule/Unit	Pattern Unit Recognizer	Created based on previous research (e.g., Clements et al., 2014)	NA
**Spatial**					
**Within**					
6	Arrange sticks in order of increasing length (for example, long, longer, longest)	Measurement - Serial Order	Length Comparer Direct	Deflorio & Beliakoff (2015)	Arrange sticks in order of increasing length

9	Cut apart a rectangle to make two squares	Recognize Shapes	Shape Recognizer - All Rectangles	Created based on previous research (e.g., Clements et al., 2014)	NA
17	Name the following shapes: circle, triangle, and square	Geometry - ID Shapes	Shape Recognizer - Typical	Deflorio & Beliakoff (2015)	Same as current
19	Measure the length of a pencil using string	Measurement - with Objects	End To End Length Measurer	Deflorio & Beliakoff (2015)	Same as current
21	Put a circular puzzle piece in the circular space	Geometry - Shape Matcher	Shape Matcher - Identical, Orientation, Sizes	Created based on previous research (e.g., Clements et al., 2014)	NA
24	Describe the properties of shapes (for example, says “It has three sides and three points so it’s a triangle.”)	Recognize Shapes	Shape Recognizer - Circle, Square, Triangle	Created based on previous research (e.g., Clements et al., 2014)	NA
26	Understand location words such as "under", "on", "next to", and "behind"	Spatial Orientation	Local Framework User	Deflorio & Beliakoff (2015)	Understand spatial words such as "under", "on", "next to", and "behind"
**Beyond**					
7	Use shapes to construct a larger geometric shape, such as using right triangles to construct a square	Geometry - ID Shapes	Parts Of Shapes Identifier	Deflorio & Beliakoff (2015)	Use pattern blocks to construct a larger geometric shape, such as using 2 right triangles to construct a square
15	Measure the angles of a triangle	Geometry - Measure Angles	NA	Deflorio & Beliakoff (2015)	Measure the angles of a triangle
28	Measure the width of a sheet of paper using a ruler (for example, uses a ruler to determine that a paper is 5 centimeters long)	Measurement - With Ruler	Length Unit Relator and Repeater	Deflorio & Beliakoff (2015)	Measure the width of a sheet of paper using a ruler

Table S2

Comparison of Confirmatory Factor Analysis Models of the Measure

Model	f	Maximum Likelihood Ratio Chi-Square [a]	NNFI [b]	CFI [c]	RMSEA [d]	AGFI [e]
			**Study 1**			
**Numeracy**						
1-factor Model	5	175.71*	0.48	0.52	0.16	0.70
Correlated 2-factor model [f]	2	77.43*	0.78	0.84	0.09	0.85
1-factor Model (Within Only)	4	48.84*	0.68	0.79	0.12	0.84
**Pattern**						
1-factor Model	5	93.25*	0.55	0.66	0.10	0.81
Correlated 2-factor model [f]	5	55.55*	0.73	0.81	0.09	0.87
1-factor Model (Within Only)	4	23.46	0.79	0.86	0.06	0.92
			**Study 5**			
**Numeracy**						
1-factor Model	5	200.85***	0.48	0.59	0.12	0.81
Correlated 2-factor model [f]	2	43.34	0.90	0.93	0.05	0.91
1-factor Model (Within Only)		20.55*	0.75	0.85	0.09	0.92
**Pattern**						
1-factor Model	4	226.08***	0.64	0.70	0.10	0.82
Correlated 2-factor model [f]	0	93.09***	0.87	0.91	0.06	0.92
1-factor Model (Within Only)	0	39.72**	0.89	0.92	0.05	0.95

Notes. *** $p < .001$ ** $p < .01$ * $p < .05$

Adequate fit is indicated by [a] Non-significant Maximum Likelihood Ratio Chi-Square, [b] NNFI ≥ 90 (Coroiu et al., 2018; Kline, 2005). [c] CFI ≥ 0.90 (e.g., Awang, 2012; Coroiu et al., 2018; Kline, 2005). [d] RMSEA ≤.08 (Coroiu et al., 2018, Browne & Cudeck, 1993). [e] AGFI ≥ .90 (Cornell Statistical Consulting Unit). [f] Items measuring similar skills were allowed to correlate (i.e., two simple arithmetic items, two magnitude comparison items, two extend pattern items, two create pattern items, and two abstract pattern items).

Table S3

Parents' responses about how they decided on their answers about early math development

Code	Definition	Example	%
Child's current ability and/or expectations for growth	Parents consider their 3- or 4-year-old child's current ability and potential development within a year or two.	"I just thought of what my daughter knows now and what I thought would be realistic for her to know in a year"	45
Experience with other children	Parents consider their experience or interaction with their own older children, other children, or their memory of their knowledge while they were children.	"I thought about what my older kids were able to do by the age of 5"<br>"...what I see kids in his class do…"	54
Benchmark or other resources	Parents consider the information they have received from schools, research articles, or other resources.	"I've thought about some articles I've read and some information that teacher friends and her pediatrician have mentioned."<br>"... what they teach at the classroom."	16
Average expectations	Parents consider what they think an average child can do by age 5 or what is realistic for 5-year-olds without specifying their source of knowledge.	"I thought about where I see my child in five years and compared that to the average child."	31

Note. Percentage of parents whose responses fit the coding scheme (n = 196). The responses of 43% of the total sample (n = 148) were too vague or otherwise did not fit the coding scheme.

Notes about the coding process: We coded parents' responses using a coding scheme (see Table S3) that was developed based on parents' responses to the same question during Study 3. Three individuals coded the data. The coders double-coded 40% of the data in three rounds and had good reliability in the final round of coding (kappa = .96) and across all three rounds (kappa = .83).
